# Ultra-broadband Reflective Metamaterial with RCS Reduction based on Polarization Convertor, Information Entropy Theory and Genetic Optimization Algorithm

**DOI:** 10.1038/srep37409

**Published:** 2016-11-22

**Authors:** Si Jia Li, Xiang Yu Cao, Li Ming Xu, Long Jian Zhou, Huan Huan Yang, Jiang Feng Han, Zhao Zhang, Di Zhang, Xiao Liu, Chen Zhang, Yue Jun Zheng, Yi Zhao

**Affiliations:** 1 Information and Navigation College, Air Force Engineering University, Xi’an 710077, China; 2 Science and Technology on Electronic Information Control Laboratory, Chendu 610036, China; 3 School of Electronic Engineering, University of Electronic Science and Technology of China, Chengdu 611731, China; 4 Department of Electronic Engineering, Tsinghua University, Beijing, 100084, China

## Abstract

We proposed an ultra-broadband reflective metamaterial with controlling the scattering electromagnetic fields based on a polarization convertor. The unit cell of the polarization convertor was composed of a three layers substrate with double metallic split-rings structure and a metal ground plane. The proposed polarization convertor and that with rotation angle of 90 deg had been employed as the “0” and “1” elements to design the digital reflective metamaterial. The numbers of the “0” and “1” elements were chosen based on the information entropy theory. Then, the optimized combinational format was selected by genetic optimization algorithm. The scattering electromagnetic fields had been manipulated due to destructive interference, which was attributed to the control of phase and amplitude by the proposed polarization convertor. Simulated and experimental results indicated that the reflective metamaterial exhibited significantly RCS reduction in an ultra-broad frequency band for both normal and oblique incidences.

In recent years, great efforts had been dedicated to the metamaterial focusing in microwave, terahertz and optical frequencies. The metamaterial inspired many applications such as acoustic cloaks, gradient index lenses, perfect absorbers, polarization rotators, and many other devices^1^,^2^,^3^,^4^,^5^,^6^. Metamaterial is a man-made artificially periodic or aperiodic structure material with the sub-wavelength unit structure^7^,^8^,^9^. As well as focusing on the metamaterials that control the near and far electromagnetic scattering fields based on the different mechanisms. Especially, the low far-field scattering such as mono-static radar cross section (MRCS) had been adequately paid attention to because of the demand of stealth for platforms. For obtaining low MRCS, perfect metamaterial absorber (PMA) with near-unity absorptivity and ultrathin structure was firstly proposed by Landy *et.al.*^10^ in 2008. It had become an important research aspect of metamaterials. Later, lots of researchers made efforts on different PMA structures to achieve broadband absorption and insensitive polarization^11^,^12^,^13^,^14^,^15^,^16^. Another way of achieving low MRCS using planar configuration was proposed by M. Paquay^17^. Based on a combination of artificial magnetic conductors (AMC) and perfect conductors in a chessboard like configuration, the planar configuration exhibited the narrow band RCS reduction. Then different combinations of several AMC structures were proposed for broadband or multiband MRCS reduction^18^,^19^,^20^,^21^. Recently, coding metamaterial and digital metamaterial have attracted more attention for significantly manipulating the electromagnetic (EM) waves^22^,^23^. In designs, the coding sequences of “0” and “1” elements are introduced to control the scattering fields. On these bases, various functionalities such as anomalous reflection, polarization conversion, scattering beam diffusion can be applied to obtain the low MRCS^24^,^25^,^26^,^27^,^28^,^29^,^30^. Importantly, metamaterials or metasurfaces could manipulate the polarization of EM waves with asymmetric transmission or reflection. Hence, they were designed as the circular polarizers or polarization rotators^31^,^32^,^33^,^34^,^35^. Aiming at manipulating the polarization, the amplitude and phase of the scattering fields have been controlled by metamaterials. More recently, the metasurface was demonstrated to achieve the multiband and broadband low MRCS based on the polarization rotation reflective surface^36^,^37^. In application, the metamaterials or metasurfaces have been used for RCS reduction of antennas^38^,^39^,^40^. However, the bandwidth of metamaterials or metasurfaces could not satisfy the application of RCS reduction for antennas. Therefore, it is a major challenge that is to extend the operating bandwidth of low RCS for the metamaterial.

In this paper, we proposed an ultra-wideband reflective metamaterial with RCS reduction based on a polarization convertor. The unit cell of the convertor manipulated a linearly polarized wave to its cross-polarized one. To design the reflective metamaterial, the proposed polarization convertor and that with rotation angle of 90 deg had been employed as the “0” and “1” elements based on the concept of digital reflective metamaterial. According to the information entropy theory, the numbers of the “0” and “1” lattices had been chosen. The combinational format of the “0” and “1” lattices was selected based on the genetic optimization algorithm. The reflective metamaterial device with similar geometry in simulation were fabricated and measured to clearly validate our design. The ultra-broadband RCS reduction for proposed reflective metamaterial were illustrated by simulated and measured results. This presented reflective metamaterial provided a more effective and reliable method to design metamaterial for ultra-broadband low scattering.

## Results

### Reflective convertor element, Number selection and Combinational format optimization

**Figure 1:**
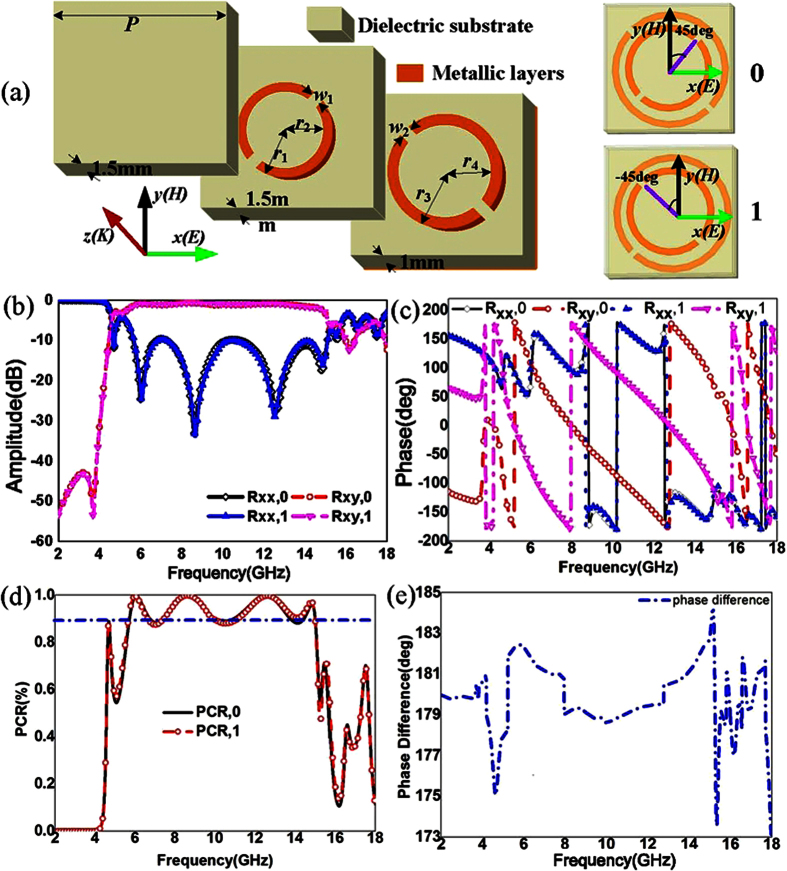
Geometry of the reflective convertor elements “0” and “1” and their reflective parameters. (**a**) Geometrical parameters of the convertor. The dimensions of the convertor are *P* = 10 mm, r_1_ = 3.42 mm, r_3_ = 4.6 mm, r_2_ = 2.92 mm, r_4_ = 4.1 mm and *w*_1_ = *w*_2_ = 0.5 mm. Elements “0” and “1” are respectively the convertors with angles of 45 deg and −45 deg. (**b**) Amplitude of reflective coefficients for elements “0” and “1” with co-polarization (xx) and cross-polarization (xy). (**c**) Phase of reflective coefficients versus frequency. (**d**) Polarization conversion ratio (PCR) of the elements “0” and “1”. (**e**) Phase difference between the element “0” and element “1”.

The reflective convertor in this paper is composed of metamaterials, which can be used for controlling the reflective parameters of scattering waves. Consequently, it is an attractive choice to achieve some interesting functionalities such as focusing, low scattering and wave bending. A reflective convertor and that with rotation angle of 90 deg have been chosen as “0” and “1” elements to design the reflective metamaterial with low RCS. The reflective convertor elements are shown in Fig. 1(a). The unit cell of proposed reflective convertor is composed of a three-layer dielectric substrate with double metallic split-rings structure and a full copper ground plane on the bottom. The dielectric substrate are all Arlon AD430 (ε_r_ = 4.3 and tanδ = 0.003), and their thicknesses are 1.5 mm, 1.5 mm and 1 mm respectively. The conductivity of copper is 5.8 × 10^7^ S/m and the thickness is 36 μm. For verifying polarization convertor, numerical simulations are performed using the software HFSS. According to the coding metamaterial, the proposed polarization convertor and that with rotation angle of 90 deg had been employed as the “0” and “1” elements. From Fig. 1(b and c), the elements of “0” and “1” effectively manipulate the reflective amplitude and phase of incidence in an ultra-wide frequency band. The polarization conversion ratio (PCR) is more than 90% for two elements as shown in Fig. 1(d) from 5.71 GHz to 15.02 GHz. Figure 1(e) shows the phase difference between elements “0” and “1”. It can be seen that the reflective convertor yields around 180 deg phase difference over the ultra-broad spectral band.

**Figure 2:**
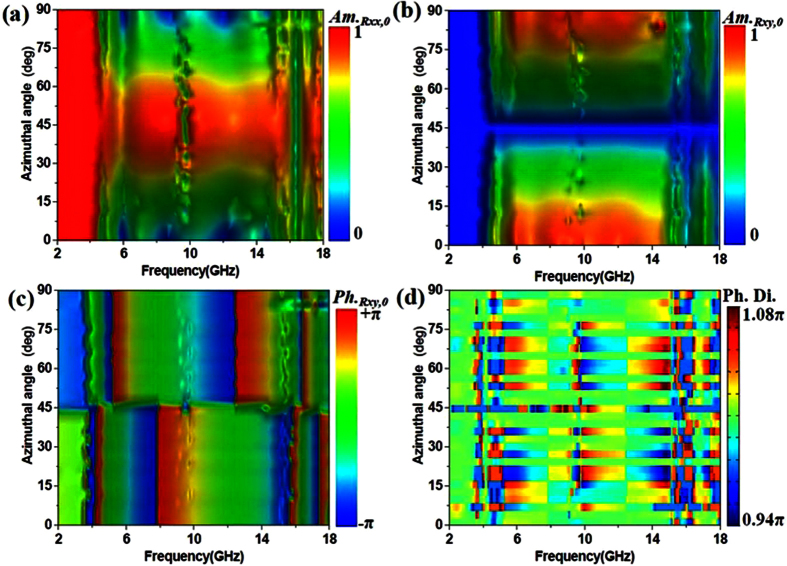
Simulated results of reflective parameters for convertor with different azimuthal angles from 2 GHz to 18 GHz. (**a**) Amplitude of reflective coefficients for element “0” with co-polarization (***Am***._***Rxx***,***0***_). (**b**) Amplitude of reflective coefficients for element “0” with cross-polarization (***Am***._***Rxy***,***0***_). (**c**) Angle of reflective coefficients for element “0” with cross-polarization (***Ph***._***Rxy***,***0***_) (**d**) Phase difference between the element “0” and element “1” with the azimuthal angle shifted from 0 deg to 90 deg.

Figure 2 shows the simulated results of reflective parameters for the proposed reflective convertor with different azimuthal angles from 2 GHz to 18 GHz. The amplitudes of reflective coefficients for element “0” (***Am***._***Rxx***,***0***_) have an obviously transformation from 0 to 1 with the co-polarized incidence. The deep spectra in Fig. 2(a) close to 10 GHz are exhibited due to the stronger coupling effects between the double metallic split-rings structures and the metallic ground. The coupling effects of double metallic split-rings structures are greatly excited at the resonance frequency. For the cross-polarization, the change of amplitudes of reflective coefficients for element “0” (***Am***._***Rxy***,***0***_) is opposite shown in Fig. 2(b). When the azimuthal angle is 45 deg, the proposed convertor exhibits the characteristics like an artificial magnetic conductors because ***Am***._***Rxx***,***0***_ is close to 1 and phase is near to zero. As shown in Fig. 2(d), the phase difference is only shifted from 0.94π to 1.08π as the azimuthal angle changes from 0 deg to 90 deg. Therefore, the azimuthal angle hardly affects the phase difference.

**Figure 3:**
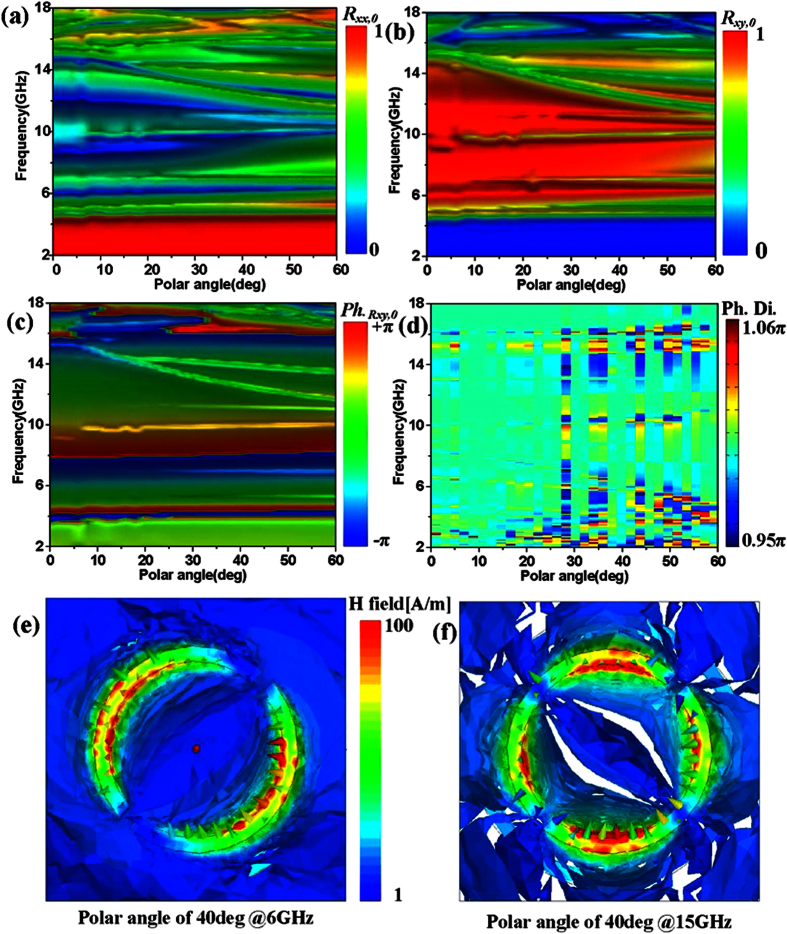
Simulated reflective parameters of unit cell versus polar angle from 0 deg to 60 deg with different frequency. (**a**) Amplitude of reflective coefficients for element “0” with co-polarization (***Am***._***Rxx***,***0***_). (**b**) Amplitude of reflective coefficients for element “0” with cross-polarization (***Am***._***Rxy***,***0***_). (**c**) Angle of reflective coefficients for element “0” with cross-polarization (***Ph***._***Rxy***,***0***_) (**d**) Phase difference between the elements “0” and “1” versus polar angle. (**e**) H-fields of proposed convertor at 6 GHz with polar angle of 40 deg. (**f**) H-fields of proposed convertor at 15 GHz with polar angle of 40 deg.

Figure 3 shows the simulated reflective properties of unit cell versus polar angle from 0 deg to 60 deg with different frequency. The bandwidth of ***Am***._***Rxx***,***0***_ ≈ 0 is slowly reduced as the polar angle shifts from 0 deg to 60 deg in Fig. 3(a). We can see that the proposed convertor performs ultra-broad bandwidth with ***Am***._***Rxy***,***0***_ ≈ 1 in Fig. 3(b). Over the frequency range from 12 GHz to 18 GHz, the phase of ***Am***._***Rxy***,***0***_ has a clear change due to the influence of the coupling effects of higher order modes between double metallic split-rings structure as the polar angle shifts from 0 deg to 60 deg. For example, the H-fields of proposed convertor with polar angle of 40 deg at 6 GHz and 15 GHz are illuminated in Fig. 3(e and f). It is observation that the H-fields change clearly with the excitation of higher order modes when the polar angle is 40 deg. This phenomenon is attributed to that the influence of higher order modes increases when the dimension of unit cell is about half wavelength of 15 GHz. When the frequency is 6 GHz, the dimension of unit cell is less than quarter wavelength of 6 GHz and the higher order modes hardly affect the performance of proposed convertor. As shown in Fig. 3(d), it is necessary to point out that the polar angle hardly affects the phase difference because that is near to π with different polar angles. According to the simulated results shown in Figs 2 and 3, the proposed metamaterial performs the wide angle polarized conversion.

**Figure 4:**
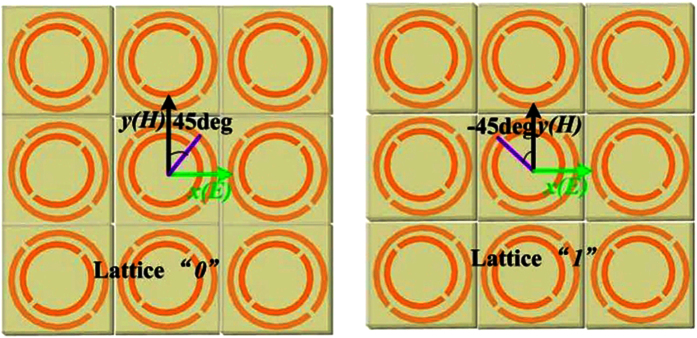
Lattices “*0*” and “*1*”. Lattice “*0*” is composed of 3 × 3 elements “0” and the lattice “*1*” is composed of 3 × 3 elements “1” to satisfy the periodic boundary in simulation. The period of the each lattice is 30 mm.

To satisfy the periodic boundary, each lattice is composed of 3 × 3 elements with the same dimension and structure. The lattices “*0*” and “*1*” as the coding sequence given in Fig. 4 can be constituted the ultra-broadband reflective metamaterial.

### Simulations and measurement

After designing the lattices “*0*” and “*1*”, the total number of the lattices is depended on the area of the reflective metamaterial. The number of lattices “*0*” and “*1*” is chosen according to the information entropy theory. It is well known that a perfect electric conductor (PEC) plane would exhibit a strong directive scattering beam for plane incident waves. For controlling the reflective scattering beam, we introduced the destructive interference based on the Snell’s law. In order to minimize the scattering fields, it is necessary to enhance the destructive interference which can be controlled by the lattices “*0*” and “*1*”. For giving a well-understood process, a coding matrix ***T*** has been introduced to describe a reflective metamaterial with *M* × *N* lattices array.


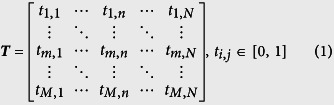


In the coding matrix ***T***, the element *t*_*m*,*n*_ is either lattice “*0*” or lattice “*1*”. Obviously, the decision that *t*_*m*,*n*_ = *0* or *t*_*m*,*n*_ = *1* is essentially random for designing the reflective metamaterial. As well, the numbers of lattice “*0*” and lattice “*1*” can’t be determined. From the information entropy theory, it is known that the information is essentially random and should be regarded as aleatory variable of non-determinism. Therefore, the information entropy can be introduced to determine the numbers of lattice “*0*” and lattice “*1*” in coding matrix ***T***.


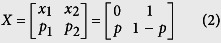


Here, assume that there are two possible answers for a random variable (such as *X*), *X* takes discrete *x*_i_ with a prior probability *p*_i_, *i* = 1, 2. The information entropy *H* can be calculated by


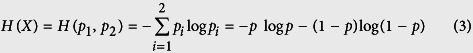


**Figure 5:**
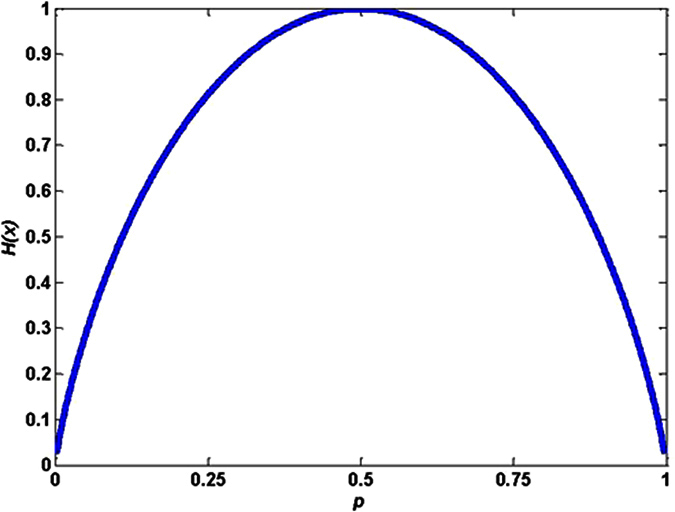
Information entropy *H* of the lattice “*0*” and lattice “*1*”.

According to equation (3), the information entropy *H* is a function of the prior probability *p*. The relationship curve between *p* and *H* is given in Fig. 5. Form Fig. 5, we can see that *H* achieves maximum value when *p* = 0.5. So the numbers of lattice “*0*” and lattice “*1*” are all (*M* × *N*)/*2.* The total scattering fields (*E*_*scattering*_) of the reflective metamaterial with the uniform plane incident waves can be regarded as superposition of the scattering wave from each basic lattice. It can be expressed as


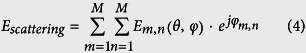


where *E*_*mn*_ is the far-field scattered pattern function of the basic lattice *t*_*m*,*n*_. *θ* and *φ* are respectively the polar angle and azimuthal angle. In order to rapid calculation, the coupling between lattices “*0*” and “*1*” can be ignored due to the configurable orthogonality. According to the array theory, *E*_*scattering*_ can be given as


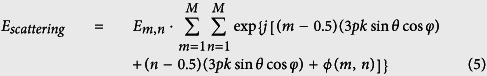


where *k* is the wave vector. *ϕ*(*m, n*) is the reflective phase of each lattice which is translated into “*0*” and “1” and the values of *ϕ*(*m, n*) are determined by the coding matrix ***T***. Hence, the total scattering fields can be depended on the pattern function of the basic lattice *E*_*m*,*n*_ and the coding matrix ***T***. It is noted that the *E*_*scattering*_ in Eq. (5) is an approximate model which does not correspond to the realistic reflective metamaterial configurations. The scattering patterns can be calculated from the approximate model.

**Figure 6:**
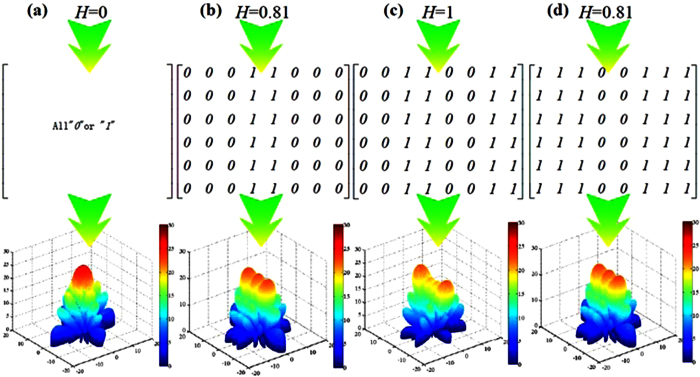
Coding matrices and their corresponding patterns with different information entropy. (**a**) *H* = 0 bit. (**b**) *H* = 0.81 bit. (**c**) *H* = 1 bit. (**d**) *H* = 0.81 bit.

For a reflective metamaterial with 6 × 8 lattices, the different coding matrices with different information entropy and their corresponding scattering patterns are given in Fig. 6 based on the lattice “*0*” and lattice “*1*”. It is observed that the scattering beam can be efficiently controlled by the reflective metamaterial when the number of lattice “*0*” is equal to that of lattice “*0*” (*H* = 1 bit) from Fig. 6(c). The reflective metamaterial with *H* = 0 bit leads to a strong reflection beam toward the normal direction as shown in Fig. 6(a). From Fig. 6(b and d), the reflective metamaterials with different coding matrices perform the same scattering pattering. This phenomenon is mainly attributed to the same information entropy and the reciprocity principle between the lattice “*0*” and lattice “*1*”.

**Figure 7:**
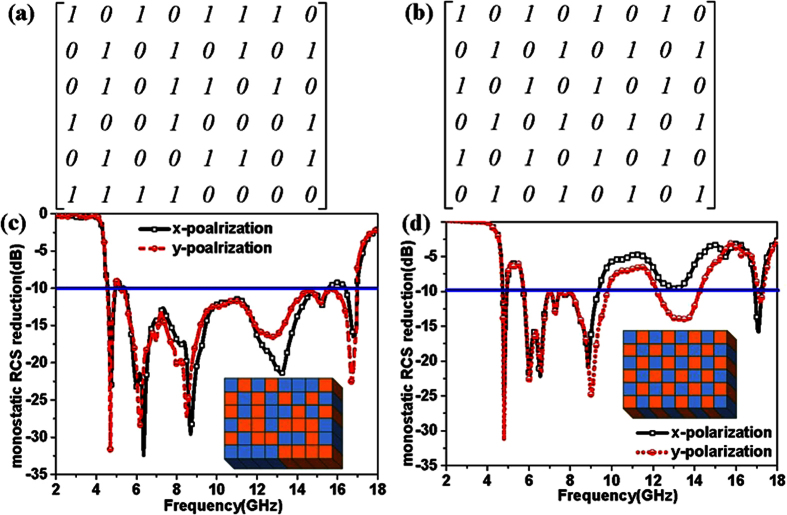
Coding matrices and their corresponding monostatic RCS reduction compared to the perfect electric conductor with same area. (**a**) Optimized coding matrix. (**b**) Uniform coding matrix (**c**) Monostatic RCS reduction with optimized coding matrix by the genetic algorithm. (**d**) Monostatic RCS reduction with uniform coding matrix.

In order to obtain ultra-broad RCS reduction, the combinational format optimization is the key for designing the reflective metamaterial. In this paper, an optimized format has been selected by the genetic algorithm (The process of the algorithm is given in the appendix A). After selection with the genetic algorithm, the optimized coding matrix *T*_6×8_ has been given in Fig. 7(a). The uniform coding matrix is given in Fig. 7(b). Correspondingly, the simulated results of RCS reduction for the reflective metamaterial are shown in Fig. 7(c and d) with optimized and uniform coding matrices. From Fig. 7(c and d), we can see that the ultra-broadband RCS reduction of the reflective metamaterial with optimized coding matrix can be obtained from 4.51 GHz to 16.99 GHz for y-polarized incidence and from 4.55 GHz to 15.19 GHz for x-polarized incidence. For the uniform coding matrix, the RCS reduction of 10 dB for the reflective metamaterial only covered the frequency from 5.72 GHz to 9.45 GHz. The difference of RCS reduction between x-polarization and y-polarization is attributed to the rectangular matrix (***T***_M,N_, M ≠ N). From Fig. 7(c), we can see that the peaks of RCS reduction are achieved for reflective metamaterial with different polarized incidences at 6.15 GHz and 16.75 GHz.

**Figure 8:**
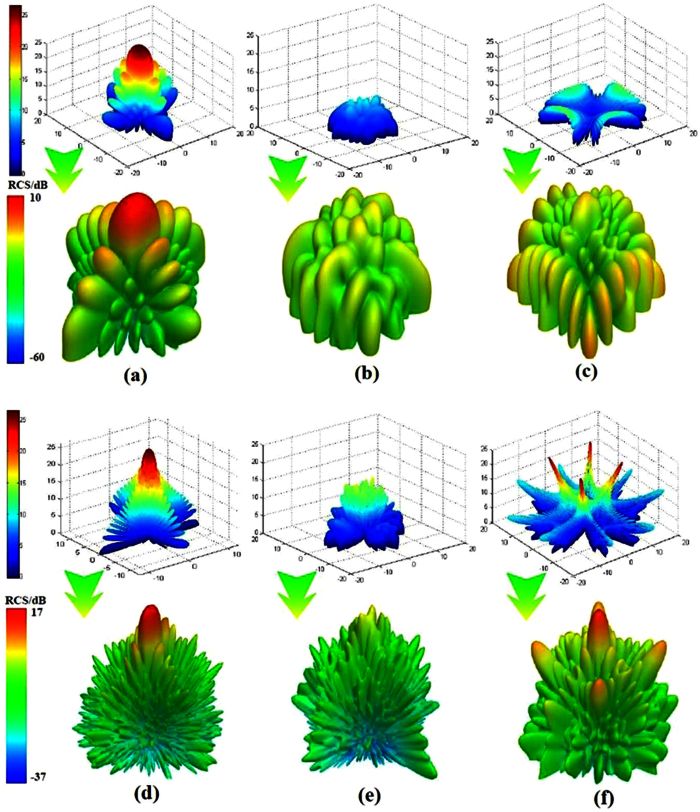
Simulated scattering patterns of PEC and reflective metamaterials with optimized coding matrix and uniform coding matrix at 6.15 GHz and 16.75 GHz for normal incidences. (**a**) Patterns of PEC at 6.15 GHz which calculated by Eq. (5) and simulated by HFSS. (**b**) Patterns of reflective metamaterial with optimized coding matrix at 6.15 GHz. (**c**) Patterns of reflective metamaterial with uniform coding matrix at 6.15 GHz which calculated by Eq. (5) and simulated by HFSS. (**d**) Patterns of PEC at 16.75 GHz. (**e**) Patterns of reflective metamaterial with optimized coding matrix at 16.75 GHz. (**f**) Patterns of reflective metamaterial with uniform coding matrix at 16.75 GHz.

The simulated scattering patterns of PEC and reflective metamaterials are demonstrated in Fig. 8 with optimized coding matrix and uniform coding matrix for normal incidences at 6.15 GHz and 16.75 GHz. Compared to the scattering patterns of PEC, the patterns of reflective metamaterials with different coding matrices obviously perform RCS reduction not only at 6.15 GHz but also at 16.75 GHz. The direction of the scattering beam has been manipulated due to the controlling of phase and amplitude by the polarization convertor. As shown in Fig. 8(b,c,e and f), it can be seen that the RCS reduction of reflective metamaterial with optimized coding matrix is more than that with uniform coding matrix at 6.15 GHz and 16.75 GHz. Therefore, the ultra-broadband RCS reduction can be obtained for the reflective metamaterials with the genetic optimized algorithm.

**Figure 9:**
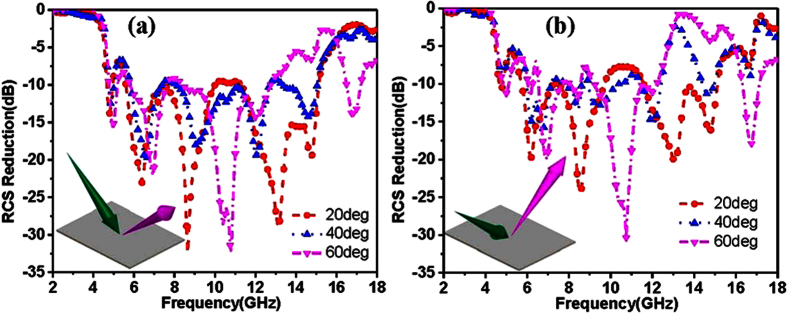
RCS reduction of the reflective metamaterials with optimized coding matrix with incident angles of 20 deg, 40 deg and 60 deg. (**a**) RCS reduction for x-polarized incidence. (**b**) RCS reduction for y-polarized incidence.

Figure 9 shows the RCS reduction of the reflective metamaterials with optimized coding matrix with incident angle of 20 deg, 40 deg and 60 deg for x-polarized and y-polarized incidences. It can be seen that the RCS reduction of reflective metamaterials can be achieved with the oblique incidences for x-polarization and y-polarization. It is noted that the RCS reduction is decreasing as the incident angle increases. Hence, we conclude that the proposed reflective metamaterials with optimized coding matrix exhibit ultra-wide band RCS reduction for normal and oblique incidences from Figs 7 and 9.

**Figure 10:**
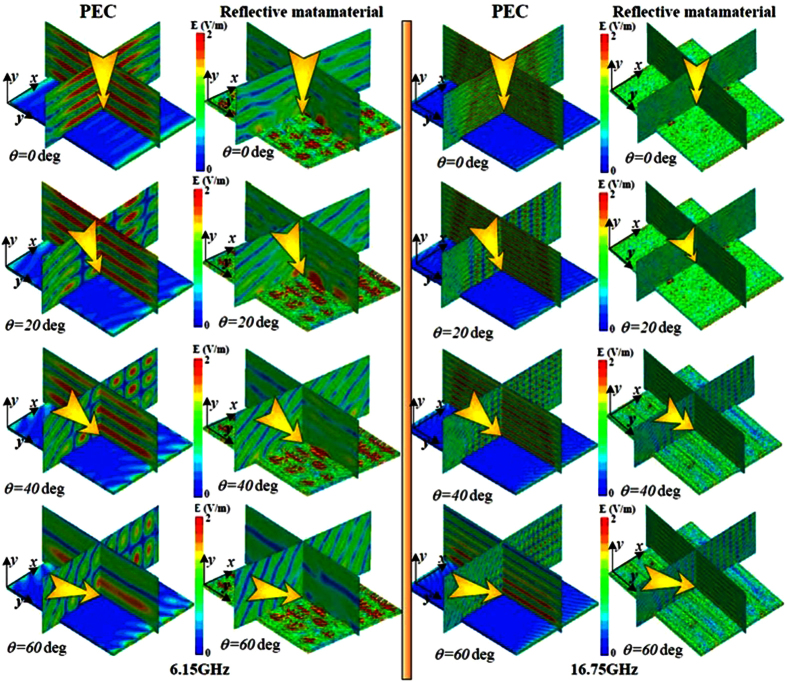
Near-fields of reflective metamaterials with optimized coding matrix and PEC at 6.15 GHz and 16.75 GHz for incident angle of 0 deg, 20 deg, 40 deg, and 60 deg. The incidence is x-polarized incident waves.

In order to validate the RCS reduction of reflective metamaterials with optimized coding matrix, the near-fields of oblique incidence are shown in Fig. 10 for x-polarization at 6.15 GHz and 16.75 GHz. It can be seen that the near scattering fields have been manipulated with incident angle not only of 0 deg, 20 deg but also of 40 deg & 60 deg. Noted that the components of scattering fields along x-axis have been remarkably manipulated by proposed reflective metamaterial due to the control of phase and amplitude by polarization convertor and the destructive interference by lattices “*0*” and “1” at 6.15 GHz and 16.75 GHz. From Fig. 10, the change tendency of the scattering fields is consistent for the x-polarized incidences as the incident angle shifts from 0 deg to 60 deg.

**Figure 11:**
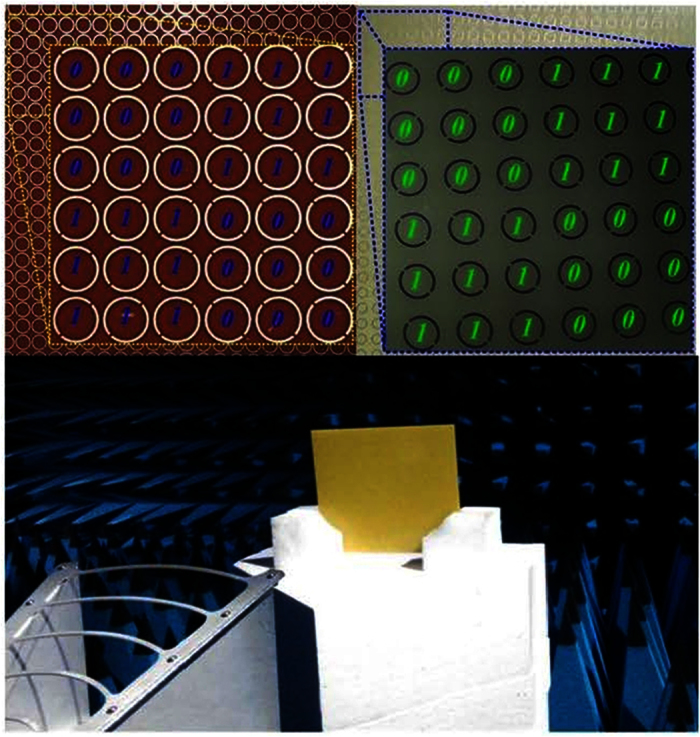
The measurement setup and fabricated reflective metamaterials device. The height of antennas and device is the same with each other. The distance is far enough to satisfy the far field requirement.

**Figure 12:**
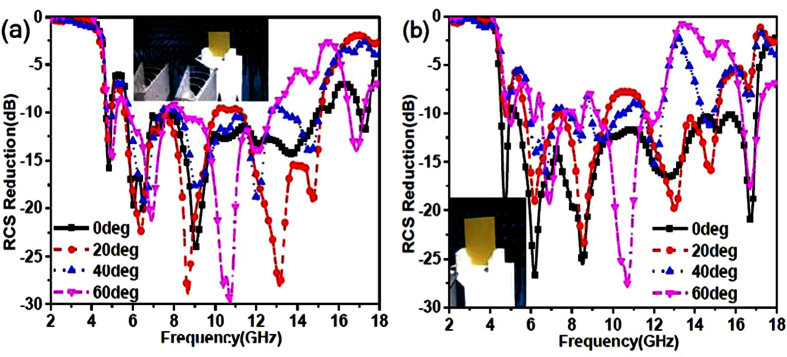
Experimental RCS reduction of the reflective metamaterials device compared to the PEC ground with the same area for incident angle of 0 deg, 20 deg, 40 deg, and 60 deg. (**a**) RCS reduction with x-polarized incidences. (**b**) RCS reduction with y-polarized incidences.

To demonstrate the reflective metamaterials, a device easily implemented using common printed circuit board method with 6 × 8 lattices was fabricated and measured by employing the free-space test method in a microwave anechoic chamber. As shown in Fig. 11, the reflective metamaterial device is given. The dielectric substrate is chosen as F4B boards with the thicknesses of 1.5 mm, 1.5 mm and 1 mm. The double metallic split-rings structure and the ground plane are made of 0.035 mm-thick copper layers. A vector network analyzer (Agilent N5230C) and two standard-gain horn antennas were used for transmitting and receiving the EM waves. The device was placed vertically in the center of a turntable to ensure that the incidence wave was similar to a plane wave in the front of device for measurement. Figure 12 shows the measured results of the reflective metamaterial device for different incident angles with x-polarized and y-polarized incidences. The ultra-broadband RCS reduction can be achieved for the reflective metamaterial device with x- and y-polarizations. The measured bandwidth of RCS reduction of 10 dB covers from 5.21 GHz to 15.09 GHz for x-polarized incidence and from 4.77 GHz to 17.08 GHz for y-polarized incident wave with incident angle of 0 deg. The different bandwidths of RCS reduction of 10 dB with x- and y-polarizations are performed due to the dissymmetrical structure of fabricated device. From Fig. 12, the broadband RCS reduction is performed due to the destructive interference of the scattering fields which is attributed to the manipulation of phase and amplitude by the reflective convertor. The comprehensive comparison between this proposed reflective metamaterial and other metamaterials in reference are given in appendix B. It is noted that the bandwidth would decrease as the incident angle shifted to 60 deg. The reflective metamaterial device performs broadband RCS reduction from 5.8 GHz to 12.2 GHz for x-polarized incidence and from 5.6 GHz to 12.1 GHz for y-polarized incident wave when the incident angle is 60 deg. The RCS reduction at high frequency is deteriorated. These physical phenomena are all attributed to the excitation of higher order modes for the reflective metamaterial at oblique incidences. At high frequency, the dimension of the unit cells special lattices is larger than the operating wavelength. The RCS reduction is achieved due to the more excitation of higher order modes and the lower excitation of basic mode for the reflective metamaterial. As shown in Figs 7, 9 and 12, good agreement can be obtained between the measurements and simulations.

## Conclusion

A valuable reflective metamaterial was designed, analyzed, fabricated and measured based on a three-layer polarization convertor. Compared to the perfect electric conductor, the ultra-broadband RCS reduction is achieved simultaneously due to the destructive interference by the polarization convertor. In the design, the polarization convertor was composed of three layers substrate with double metallic split-rings structure and a metal ground plane. The polarization convertor and that with rotation angle of 90 deg had been employed as the “0” and “1” elements to design the digital reflective metamaterial. The information entropy theory and genetic optimization algorithm were used for choosing and selecting the numbers and combinational format of elements. Two reflective metamaterial based on the lattices array with different coding matrices were compared with each other. From the comparison, we observed that the more RCS reduction and wider bandwidth could be obtained by the information entropy theory and genetic optimization algorithm. A device with 6×8 lattices was easily implemented using common printed circuit board method. Experimental results were completely consistent with those in simulation. The results indicated that the metamaterial device performed ultra-wide band RCS reduction.

## Methods

In our designing, the scattering characteristics are measured in an anechoic chamber for making good RCS measurement over the frequency range from 2 GHz to 18 GHz. When it is for the normal incident waves, two identical horn antennas are employed and placed adjacently in front of the fabricated devices with the distance more than 3 m to support far-field test. The incidence can be approximated as plane wave due to the distance. Two identical horn antennas are used as the transmitting and receiving antennas, which are connected to the two ports of the Agilent N5230C VNA. For measuring the RCS of devices, these horn antennas as the receiver and transmitter are designed to operate in continuous waves. It is noted that all of these along with the capability to transmit and receive pulses of very short duration. In measurement, the gate-reflect-line calibration in time-domain analysis kit of VNA is used to experimentally verify and improve the testing capability. When it is for the oblique incidence, the horn antennas are set as x-polarization and placed with a same angle with respect to the normal direction of the fabricated devices. In order to achieve different polarized incidences, the fabricated devices are rotated around their central axis to measure RCS.

## Additional Information

**How to cite this article**: Li, S. J. *et al*. Ultra-broadband Reflective Metamaterial with RCS Reduction based on Polarization Convertor, Information Entropy Theory and Genetic Optimization Algorithm. *Sci. Rep.* **6**, 37409; doi: 10.1038/srep37409 (2016).

**Publisher's note:** Springer Nature remains neutral with regard to jurisdictional claims in published maps and institutional affiliations.

## Supplementary Material

Supplementary Information
