# Supplementary material for: Ultra-broadband Reflective Metamaterial with RCS Reduction based on Polarization Convertor, Information Entropy Theory and Genetic Optimization Algorithm
Source: Sci Rep. 2016 Nov 22;6:37409. doi: 10.1038/srep37409 (PMC5118813; doi:10.1038/srep37409)
Supplement: Supplementary Information [file srep37409-s1.doc]

Supplementary Information for

**Ultra-broadband Reflective Metamaterial with RCS Reduction based on Polarization Convertor, Information Entropy Theory and Genetic Optimization Algorithm**

Si Jia Li^1, *^, Xiang Yu Cao^1, *^, Li Ming Xu^2^, Long Jian Zhou^3^, Huan Huan Yang^1, 4^, Jiang Feng Han^1^, Zhao Zhang^1^, Di Zhang^1^, Xiao Liu^1^, Chen Zhang^1^, Yue Jun Zheng^1^, and Yi Zhao^1^

1Information and Navigation College, Air Force Engineering University, Xi’an 710077, China

2Science and Technology on Electronic Information Control Laboratory, Chendu 610036, China

3School of Electronic Engineering, University of Electronic Science and Technology of China, Chengdu 611731, China

4Department of Electronic Engineering, Tsinghua University, Beijing, 100084, China

*These authors contributed equally to this work.

+Correspondence and requests for materials should be addressed to S.J. L. (email:lsj051@126.com) or X. Y. C. (gjgj9694@163.com)

**This file includes:**

Supplementary Appendix A

Supplementary Appendix B

***Appendix A***

***Genetic Optimization Algorithm***

The genetic optimization algorithm had been applied in our designs. The flowchart showing the operation of the genetic algorithm is given in figure 1. In the optimized processes, the objective function can be expressed as

(1)

The constraint equations are given as follows.

(2)

Where $f("0")$ and $f("1")$ represent the number of basic lattices “*0*” and “*1*”, respectively.


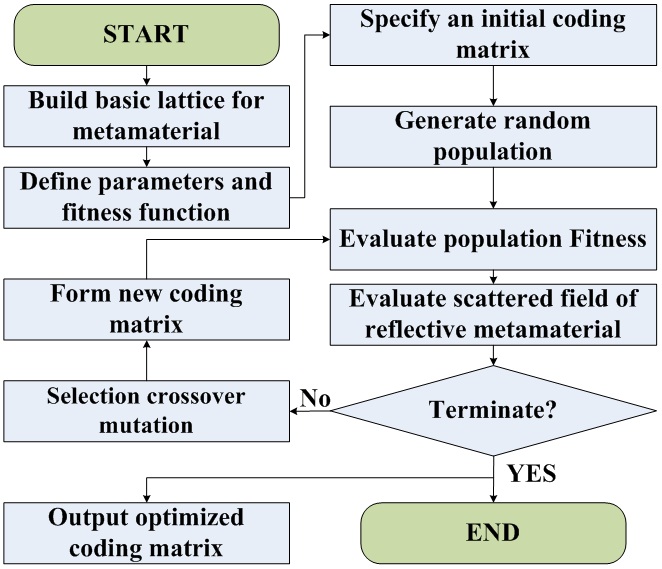


**Figure 1. Flowchart showing the operation of the genetic algorithm.**

The optimized results are shown as follows. After selection with the genetic algorithm, the optimized coding matrix *T*_6×8_ has been given in figure 2(a). Correspondingly, its simulated results of RCS reduction for the reflective metamaterial are shown in figures 2(b). We can see that the ultra-broadband RCS reduction of the reflective metamaterial with optimized coding matrix can be obtained from 4.51GHz to 16.99GHz for y-polarized incidence and from 4.55GHz to 15.19GHz for x-polarized incidence. The difference of RCS reduction between x-polarization and y-polarization is attributed to the rectangular matrix(***T***_M,N_, M≠N). From figure 2(b), we can see that the peaks of RCS reduction are achieved for reflective metamaterial with different polarized incidences at 6.15GHz and 16.75GHz.

**Figure 2. The optimized Coding matrix and its corresponding monostatic RCS reduction compared to the perfect electric conductor with same area.** (a) Optimized coding matrix. (b) Monostatic RCS reduction with optimized coding matrix by the genetic algorithm.

***Appendix B***

***Comparison of Reflective Metamaterials***

For comprehensive comparison, the relative volume *Rv* of a unit metamaterial cell has been introduced and is defined as ^40^:

*Rv=Tv*/(λ_0_)^3^ (3)

where *Tv* is the total volume of a unit cell, λ_0_ is the wavelength of center frequency for bandwidth with RCS reduction larger than 10dB. In order to synthesize the relative bandwidth *BW* of RCS reduction larger than 10dB for these metamaterials in reference and their volume of a unit cell, we have evaluated the cost-efficient bandwidth *BW_CE_* defined as ^40^

*BW_CE_= BW*/*Rv*  (4)

In order to better understand the performance of the proposed reflective metamaterial compared against existing metamaterials reported in literatures, we list the frequency range with RCS reduction of 10dB at normal incidence, length of a cell, thickness and cost-efficient bandwidth in Table I for the existing wideband metamaterial with RCS reduction including the one presented in this paper.

According to Table I, we can see that our proposed reflective metamaterial performs the most cost-efficient bandwidth *BW*_CE_ (62.58) of a unit cell. We can also observe that our proposed reflective metamaterial exhibits ultra-broadband RCS reduction with a relative bandwidth of 97.5% from 5.2GHz to 15.1GHz. It is noted that an ultra-broadband polarization rotation could be achieved for reflective surfaces in [36], but the metamaterial array with common coding matrix could not obtain the ultra-broadband RCS reduction due to the larger structure of lattices. Therefore, the genetic optimization algorithm is important to design the coding matrix to design the broadband low RCS reflective metamaterial.

*Table I Comparison between proposed reflective metamaterial and metamaterials in references*

|  | *Frequency range* | *Length of cell* | *Thickness* | *Cost efficient bandwidth* |
| --- | --- | --- | --- | --- |
| Ref.[21] | *7GHz ~13GHz* | *12mm* | *4mm* | *28.17* |
| Ref.[27] | *8GHz~18GHz* | *10mm* | *3mm* | *31.23* |
| Ref.[36] | *Multi-band* | *12mm* | *3mm* | *------* |
| Ref.[37] | *5.8GHz~6.6GHz* | *10mm* | *3mm* | *48.45* |
| *This Paper* | *5.2GHz~15.1GHz* | *10mm* | *4mm* | *62.58* |
